# Atrial Impairment as a Marker in Discriminating Between Takotsubo and Acute Myocarditis Using Cardiac Magnetic Resonance

**DOI:** 10.1097/RTI.0000000000000650

**Published:** 2022-04-05

**Authors:** Riccardo Cau, Christian Loewe, Valeria Cherchi, Michele Porcu, Pierluigi Ciet, Jasjit S. Suri, Luca Saba

**Affiliations:** * Department of Radiology, Azienda Ospedaliero Universitaria (A.O.U.), Cagliari, Italy; † Division of Cardiovascular and Interventional Radiology, Department of Bioimaging and Image-Guided Therapy, Medical University of Vienna, Vienna, Austria; ‡ Erasmus MC, Rotterdam, The Netherlands; § Stroke Monitoring and Diagnosis Division, AtheroPoint(tm) Roseville, CA

**Keywords:** atrial strain, Takotsubo, cardiac magnetic resonance, myocarditis

## Abstract

**Purpose:**

The purpose of this study was to comprehensively compare the left and right atrium strain and strain rate (SR) parameters by cardiac magnetic resonance (CMR) between patients with Takotsubo (TS) and patients with acute myocarditis (AM).

**Materials and Methods:**

We retrospectively enrolled 3 groups of patients: TS (n=18), AM (n=14), and 11 healthy subjects. All the patients had complete CMR data for features tracking assessment.

Differences in reservoir, conduit strain (ε_e_), conduit strain rate (SRe), and booster phase of biatrial strain were analyzed between the groups using analysis of variance and multivariate analysis of covariance analyses. Intraobserver and interobserver reproducibility was assessed for all strain and SR parameters using intraclass correlation coefficients and Bland-Altman analysis.

**Results:**

Atrial strain was feasible in all patients and controls. In TS, left atrium (LA) reservoir strain (ε_s_), reservoir SR, ε_e_, and SRe were significantly lower compared with the other groups (*P*=0,001 for all). multivariate analysis of covariance analysis showed association of these parameters after correction for age and sex, while LA booster deformation (ε_a_ and SRa) strain parameters were preserved. LA SRe proved to have excellent sensitivity in differentiating patients with TS from those with AM (areas under the curves of 0.903, 95% confidence interval: 0.81-0.99).

Biatrial strain and SR parameters showed good (excellent) intraobserver and interobserver reproducibility (ranged between 0.61 to 0.96 and 0.50 to 0.90, respectively).

**Conclusion:**

Compared with AM, patients with TS showed significantly decreased LA reservoir, conduit strain, and SR parameters. Therefore, LA strain assessment may have a role in discriminating between TS and AM.

## KEY POINTS

- Cardiac magnetic resonance (CMR) feature tracking (CMR-FT) represents a feasible and reproducible tool to assess atrial strain.
- Takotsubo syndrome (TS) patients showed left atrium (LA) impairment in comparison with the acute myocarditis (AM) group.
- CMR-FT may be a novel parameter in discriminating between TS and AM, providing new insights into the development of Takotsubo cardiomyopathy

## INTRODUCTION

Acute chest cardiac pain represents a common symptom in daily clinical practice and can be caused either by ischemic or nonischemic disease, whereas early detection of an ST elevated myocardial infarction (STEMI) is usually possible even in a preclinical setting, further differential diagnosis, in cases where an STEMI was ruled out, remains challenging. Discriminating between different nonischemic causes of acute chest pain, namely between TS and AM, represents a common diagnostic dilemma. Although clinical presentation with a possible trigger as well as different demographic data may be different between these entities, clinical phenotypes of TS may closely resemble AM, which includes beyond chest pain, dyspnea, and syncope besides similar cardiac signs, laboratory, and electrocardiography changes.^1^ In particular, TS is a well-recognized cardiomyopathy characterized by a pattern of left ventricular (LV) dysfunction, mainly presenting as apical ballooning and hyperkinesis of basal segments.^1^ Less common variants can involve mid-ventricular, basal, focal LV segments or the whole LV or both ventricles (apical LV and right ventricle [RV]), or just the RV.^1^,^2^ Recently, Stieirmaier et al^3^ reported a transient deterioration of LA strain parameters during acute/subacute phases of TS using CMR.

These wall motion abnormalities and clinical symptoms are usually transient with complete recovery within several weeks.^3^ Prognosis of TS is in most of the cases favorable, but recent studies have shown possible complications and death.^4^

Because of different clinical course, management, and outcome between TS and AM, differential diagnosis is crucial.

CMR has become the reference standard in the evaluation of cardiac function and morphology in several clinical settings and diseases.^5^ In addition, CMR has demonstrated high ability to diagnose and characterize acute and chronic myocardial diseases, and currently CMR is the gold standard to diagnose AM. Diagnostic accuracy in detection and rule out of AM is of special importance in the acute setting, as AM represents an exclusion criterion for TS according to current international diagnostic criteria.^6^–^8^

Recently, myocardial strain analysis has been used for ventricle function assessment using CMR,^9^,^10^ showing a potential role to discriminate between TS and AM.^11^ CMR-FT analysis allows assessment of regional myocardial abnormalities as well as detection of compensatory increase in other strain parameters.^11^ Besides ventricular function evaluation, atrial function also has been investigated using CMR-FT.^12^,^13^

The atria play a key role in maintaining left ventricular filling. Several articles have highlighted a significant pathophysiological contribution of atria in different cardiovascular diseases, including TS.^3^,^14^–^16^ Backhaus and colleagues reported an alteration of LA strain measurements in TS patients during the acute phase of the disease. In the setting of myocarditis, an impairment of LA strain measurements as well as an involvement of the right atrium have been described.^15^,^16^ However, little is known about the difference of atrial mechanism in the pathophysiology of these 2 entities. Consequently, the purpose of this study was to evaluate LA and right atrium (RA) strain as an alternative marker to discriminate between TS and AM using CMR.

## MATERIALS AND METHODS

### Study Population

In this retrospective single-center study we searched in our database all patients who underwent CMR between March 3, 2017 and February 7, 2021 and with a suspected diagnosis of AM or apical ballooning TS based on clinical parameters and CMR findings.

TS diagnosis was made using current definition reported in the Position Statement of the European Society of Cardiology Heart Failure Association.^7^ Criteria include regional wall motion abnormalities not limited to a single epicardial vascular distribution usually preceded by a stressful trigger, an absent of culprit atherosclerotic coronary disease assessed by invasive catheterization, new ECG abnormalities, elevated serum natriuretic peptide and small increase in cardiac troponin, and recovery of LV dysfunction at follow-up.

The diagnosis of AM was made clinically according to the current guidelines reported in the Position Statement of the European Society of Cardiology Working Group on Myocardial and Pericardial Diseases.^17^ Endomyocardial biopsy was not performed. Some of the patients who under analysis were published in our previous works.^11^,^18^ Exclusion criteria included: subjects younger than 18 years old, contraindication to CMR (implantable devices, severe claustrophobia), or a history of renal disease with a current eGFR <30 mL/min/1.73 m^2^, and coronary artery disease.

The control group comprised healthy subjects who had CMR to exclude scar-related ventricular tachycardia without known cardiovascular risk factors, and had negative studies, were used as negative controls. Informed consent was waived because of the retrospective nature.

### CMR Acquisition

CMR scans were performed after hospital admission for acute chest pain and/or dyspnea using a 1.5  T scanner system (Philips Achieva DStream, Philips Healthcare, Best, the Netherlands). Eight-channel anterior cardiac coil arrays were used. Cine CMR examinations were electrocardiogram triggered and performed during breath-hold maneuver.

Thirty phases were derived for each cardiac cycle. The CMR protocol included functional sequences, such as cine bright blood steady-state free precession (SSFP) on the short axis and long axes (2 chambers, 3 chambers, and 4 chambers); and morphologic and tissue characterization sequences, such as T2 Short Tau Inversion Recovery (STIR) on both short and long axes, precontrast and postcontrast T1 mappings, T2 mapping, and late gadolinium enhancement (LGE) acquisitions. LGE imaging was performed 10 to 12 minutes after contrast media injection (Gadovist, Bayer Healthcare, Berlin, Germany) at a dose of 0.15 mL per kilogram body weight using phase-sensitive inversion recovery sequences acquired in both short and long axes. The correct inversion time was determined using the Look-Locker technique.

### CMR Image Postprocessing

A commercially available software, Circle CVI42 (CVI42, Circle Cardiovascular Imaging Inc., Calgary, Canada), was used for CMR-FT data analysis. CMR-FT analyses of atrial deformation were conducted offline. LA and RA endocardial borders were manually traced on the long-axis view of the cine images when the atrium was at its minimum volume. In particular, 4, 3, and 2-chamber views were used to derive LA longitudinal strain. Atrial appendage and pulmonary veins were excluded from segmentation. RA longitudinal strain was based on the 4-chamber view only. After manual segmentation, the software automatically tracked the myocardial borders throughout the entire cardiac cycle. The quality of the tracking and contouring was visually validated and manually corrected by a radiologist with 3 years of experience in cardiac imaging. There are 3 peaks in the strain curve, including reservoir, conduit, and booster strain. Accordingly, their corresponding strain rate (SR) parameters were included.

For intraobserver analysis, 1 observer (R.C.), with 3 years of experience in cardiovascular imaging, performed the SR analysis, repeating all measurements twice 1 month apart in random order to avoid recall bias. For interobserver analysis, a second blinded observer (G.C.), with 2 years of experience in cardiovascular imaging, performed the SR analysis in a random set of 15 patients and healthy subjects. Both observers were blinded to all clinical data, prior test results, and diagnosis as well as to the interpretation of the other observer.

### Statistical Analysis

Continuous variables are presented as mean± SD. Kolmogorov-Smirnov tests were used to check continuous variables for normal distribution. Comparisons of continuous data were performed using independent-samples *t* test or Mann-Whitney *U* test analysis. Categorical variables were compared using χ^2^ or Fisher exact test, according to data distribution.

Comparisons between groups were performed using the 1-way analysis of variance for continuous variables with normal distributions, and the Kruskal-Wallis test for continuous variables with skewed distributions. A post hoc Tukey multiple comparison test was performed to look for statistically significant differences among each group. A general linear model analysis was performed including age and sex as covariates (multivariate analysis of covariance).

A receiver-operating characteristic (ROC) analysis was performed to calculate optimal thresholds and areas under the curves (AUCs). The Youden index was used to depict optimal cutoff values from the ROC curves. Sensitivities and specificities were calculated for these cutoff values with 95% confidence intervals.

Intraobserver and interobserver variability were assessed by intraclass correlation coefficients and Bland-Altman analysis. Correlation was assessed using the Pearson *r* and Spearman rho coefficient according to data distribution. A *P*-value <0.05 was considered statistically significant. All statistical analyses were performed using IBM SPSS Statistics version 22 (SPSS Inc., Chicago, IL).

## RESULTS

### Patient Demographics and CMR Parameters

We included 18 patients with TS (17 females, mean age 68.7 ±SD 10 y.), 14 patients with AM (6 females, mean age 43.2 ±SD 15.9 y), and 11 healthy subjects (7 females, mean age 49.8 ±SD 9.2 y). One patient with the diagnosis of TS had to be excluded due to insufficient image quality.

Baseline characteristic and CMR parameters of the enrolled patients are summarized in Table 1.

**TABLE 1:** Comparison of Demographics and CMR Findings in AM and TS

	TS	AM	Control	*P*
Age (y)	68.7±10	43.2±15.9	49.8±9.2	**0.001**
Female	17/18 (94%)	5/14 (35%)	7/11 (63%)	**0.001**
LVEF	58.7±8.9	58.2±5	59.2±4.9	0.9
RVEF	59.6±5.8	57.2±4.9	55.6±2.9	0.06
LGE g	0	12.27±9.01	0	—
LGE %	0	11.62±9.51	0	—
Troponin	2936±2446	3270±1670	0	—
LA ε_s_	24.8±5.9	30.1±7.2	35.6±3.9	**0.001**
LA SRs	1.1±0.3	1.4±0.4	1.5±0.2	**0.001**
LAε_e_	10.6±4.4	16.3±6.2	21.5±4.9	**0.001**
LA SRe	−0.9±0.4	−1.9±0.7	−1.8±0.4	**0.001**
LAε_a_	14.7±6.2	12.3±3.8	13.2±2.2	0.38
LA SRa	−1.65±0.5	−1.5±0.4	−1.73±0.3	0.45
RA ε_s_	35.9±22.6	28.2±2.2	38.7±9.1	0.25
RA SRs	1.9±0.6	−1.6±0.5	2.1±0.7	0.28
RAε_e_	21.3±15.1	17.8±7.3	24.3±9.8	0.38
RA SRe	−2.2±1	−1.4±0.3	−1.6±0.8	0.07
RAε_a_	14.9±8.7	9.7±5.5	13.4±4.8	0.11
RA SRa	−2±1.2	−1.6±1	−1.4±0.6	0.6

Bold values indicate statistically significant.

Values are represented as mean±SD.

LVEF indicates left ventricle ejection fraction; RVEF, right ventricle ejection fraction.

### Feasibility of Biatrial CMR-FT

CMR scans were performed after admission with a mean delay of 4.1 ±SD 2.6 days.

CMR-FT of biatrial myocardium strain could be assessed and analyzed successfully in all patients. Tracking quality was sufficient in all cases, based on visual checking and manual corrections.

### CMR-FT of Both Atria

Changes in RA and LA deformation parameters are reported in Table 1. LA reservoir and conduit functions demonstrated a significant difference between the groups under analysis (LA ε_s_: *P*=0.001, LA SRs: *P*=0.001, LAε_e_: *P*=0.001 and LA SRe: *P*=0.001).

**FIGURE 1:**
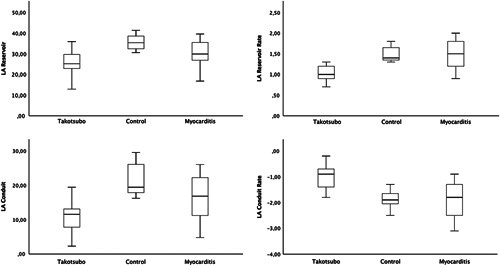
Box and Whisker plots representing the difference of left atrial strain and strain rate parameters between Takotsubo, controls and acute myocarditis.

Comparison of LA strain analysis between groups showed significant differences as shown in Table 2 and Figure 1. Tukey post hoc test revealed that reservoir strain (LA ε_s_: *P*=0.04), LA reservoir SR (LA SRs: *P*=0.007), LA conduit strain (LAε_e_: *P*=0.01), and LA conduit SR (LA SRe: *P*<0.001) were significantly lower in the TS group compared with AM and healthy groups. Conversely, LAε_a_, and RA strain parameters did not show any significant difference between the groups. Multivariate analysis of covariance analysis confirmed that the association of LA deformation parameters were independent of gender and age (Table 3).

**TABLE 2:** Multiple Comparison Tukey Post Hoc Test Between Different Groups and LA Strain Parameters

	TS vs. AM	TS vs. Control	AM vs. Control
LA ε_s_	**0.04**	**<0.001**	0.07
LA SRs	**0.007**	**0.003**	0.8
LAε_e_	**0.01**	**<0.001**	**0.04**
LA SRe	**<0.001**	**<0.001**	0.9
Age (y)	**<0.001**	**0.001**	0.37
Sex	0.13	**0.01**	0.2

Bold values indicate statistically significant.

Values are represented as mean±SD.

**TABLE 3:** Multivariate Analysis of Covariance Analysis

	Age (y)	Sex
LA reservoir strain	0.81	0.54
LA reservoir strain rate	0.09	0.14
LA conduit strain	0.19	0.78
LA conduit strain rate	0.07	0.34

### Association With Clinical and CMR Parameters

LA conduit strain and SR measurements demonstrated the highest correlation with left ventricle ejection fraction (*r*=0.396, *P*=0.009; *r*=−0.422, *P*=0.005, respectively). Correlations between LA reservoir strain and SR parameters are weak (*r*=0.312, *P*=0.019; *r*=−0.22, *P*=0.032, respectively).

There was no other statistically significant correlation between atrial strain measurement and left ventricle ejection fraction.

LA and RA strain functions did not demonstrate any significant correlation between the extent of LGE (expressed in both percent and grams) and atrial strain parameters in AM patients. Finally, there was no statistically significant correlation between troponin values and atrial strain measurement.

### ROC Analysis

**FIGURE 2:**
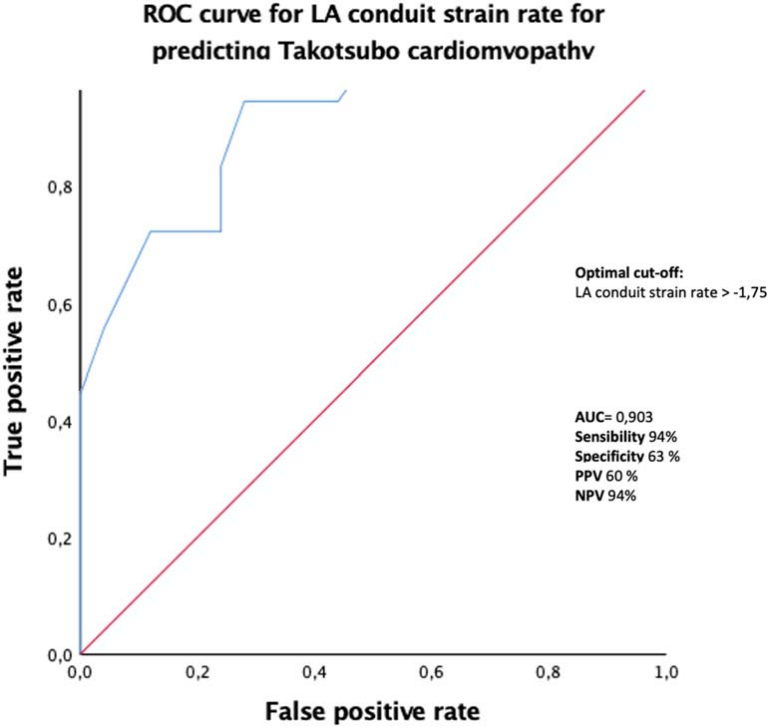
ROC curves for LA conduit strain rate to identify patients with Takotsubo. NPV indicates negative predictive vale;PPV, positive predictive value.

LA SRe proved to have excellent sensitivity in differentiating patients with TS from those with AM (AUCs of 0.903, 95% confidence interval: 0.81-0,99). Optimal cutoff for LA SRe values to identify TS was >−1,75 with sensitivity, specificity, positive predictive value, and negative predictive value of 94%, 63%, 60%, and 94%, respectively (Fig. 2).

### Interobserver and Intraobserver Analysis

**FIGURE 3:**
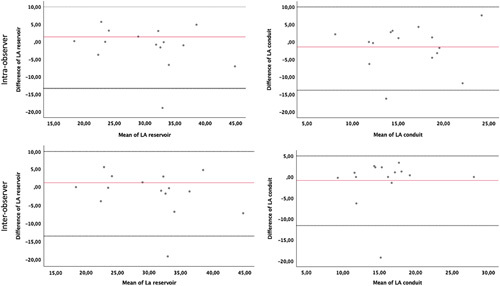
Bland-Altman plots for intraobserver and interobserver reproducibility of LA reservoir and conduit strain.

Intraobserver and interobserver intraclass correlation coefficients coefficients ranged between 0.61 to 0.96 and 0.50 to 0.90, respectively, as shown in Table 4. Bland-Altman plots showed no systematic errors and minimal differences for LA reservoir and conduit strain as shown in Figure 3.

**TABLE 4:** Intraclass Correlation Coefficients for Intraobserver and Interobserver Reproducibility of Atrial Strain and SR Parameters

	Intraobserver	Interobserver
LA ε_s_	0.85 (0.45-0.93)	0.81 (0.45-0.92)
LA SRs	0.93 (0.69-0.96)	0.89 (0.69-0.96)
LAε_e_	0.90 (0.61-0.93)	0.85 (0,57-0,95)
LA SRe	0.85 (0.57-0.95)	0>86 (0.51-0.94)
LAε_a_	0.84 (0.52-0.94)	0.78 (0.50-0.91)
LA SRa	0.61 (0.36-0.76)	0.57 (0.37-0.75)
RA ε_s_	0.87 (0.61-0.96)	0.87 (0.61-0.95)
RA SRs	0.80 (0.41-0.93)	0.80 (0.41-0.93)
RAε_e_	0.84 (0.55-0.95)	0.73 (0.37-0.90)
RA SRe	0.50 (0.48-0.83)	0.50 (0.48-0.83)
RAε_a_	0.84 (0.52-0.94)	0.82 (0.50-0.91)
RA SRa	0.61 (0.14-0.87)	0.58 (0.38-0.78)

## DISCUSSION

**FIGURE 4:**
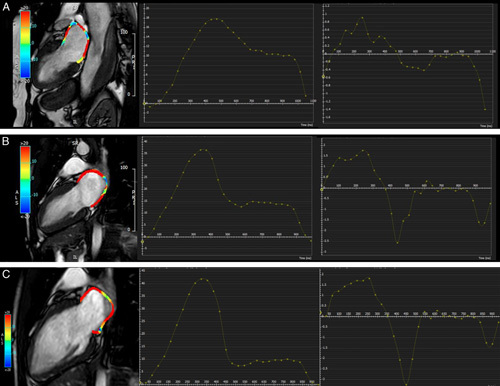
Contouring for left atrium strain and strain rate parameters. The figure showed a representative image of left atrial strain from the 2-chamber view using CMR-FT in a TS patient (A), in control subject (B), and in AM patient (C) with the corresponding strain and strain rate parameters.

**FIGURE 5:**
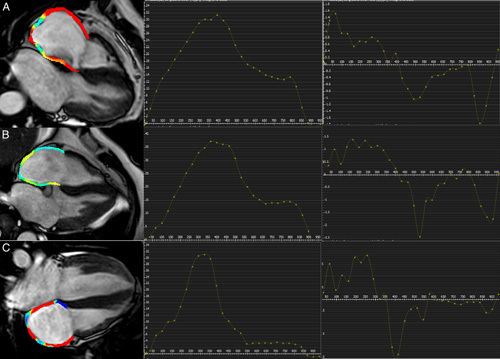
Contouring for right atrium strain and strain rate parameters. The figure shows a representative image of left atrial strain from the 4-chamber view using CMR-FT in a TS patient (A), in control subject (B) and in AM patient (C) with the corresponding strain and strain rate parameters.

We investigated the feasibility and diagnostic value of atrial strain assessment using CMR to discriminate patients with TS and AM. Compared with patients with AM and the control group, patients with TS demonstrated a significantly lower LA strain, with dysfunction of both reservoir and conduit phases of left atrium function. Figures 4 and 5 show a representative example of the left and the right atrial strain, respectively. Moreover, LA conduit SR demonstrated an excellent AUC to discriminate TS with a sensitivity of 94% and specificity of 44%.

Eitel et al^19^ reported specific CMR criteria for TS diagnosis that include a combination of typical contraction pattern, edema, and absence of LGE. Recently, ventricular myocardial strain has been evaluated as an additional useful diagnostic marker in patients with TS.^11^,^20^,^21^

Despite CMR is excellent both functional and morphological studies aimed to assess typical regional wall motion abnormalities and reversible and irreversible myocardial injuries.^5^,^11^,^22^ TS is often misdiagnosed due to a nonspecific clinical manifestation which could resemble other cardiovascular diseases, such as acute coronary syndrome and myocarditis.^1^,^23^

Our results showed that atrial strain assessed on routinely acquired SSFP cine images could help clinicians for the challenging differential diagnosis of TS, as an alternative marker beyond the traditional CMR parameters. In particular, we found a different atrial strain impairment between TS and AM patients.

The atria have a key role in maintaining ventricular filling and can be subdivided into 3 consecutive phases: reservoir, conduit, and booster. Over the past decade, the focus has been on LA enlargement,^24^ but LA size does not provide a complete overview of the LA function during the cardiac cycle. In this scenario, atrial strain analysis enables to overcome usual limitations of the sole use of LA volumetric measurement.

Recently, atrial deformation has been recognized in several cardiovascular diseases as an important marker of adverse cardiovascular events and there is a growing body of evidence that LA and RA deformation are sensitive quantitative parameters in early stage diseases.^14^,^25^

Our findings showed significantly lower LA ε_s_, LA SRs, LAε_e_, and LA SRe values in TS patients compared with a cohort of myocarditis patients. On the other hand, LAε booster strain showed an opposite trend toward higher values in TS patients, although this difference was not statistically significant. Change in RA strain and SR parameters demonstrated a similar trend of LA, although the difference between the groups under analyses was higher for LA strain.

The observed values of atrial strain in patients with TS are in line with the current literature.^26^ A potential explanation of atria dysfunction in TS patients has been recently suggested.^26^ The role of diastolic impairment in TS has been evaluated in a few studies so far, highlighting a transient LA impairment with recovery during the follow-up. Stiermar and colleagues reported an improvement in LA reservoir strain parameters from 42% during the acute phase to 51% at follow-up. Similar trends were observed for conduit and booster phases.^3^ The result of our study suggested a potential role of diastolic impairment in TS pathophysiology, highlighting atrial conduit rate parameters as a sensitive diagnostic tool in discriminating between TS and AM.

Truong et al^12^,^13^ have shown that CMR-FT had good intraobserver and interobserver reproducibility for the analysis of atrial deformation in both LA and RA. Quantitative parameters used in our study are robust, with good to excellent intraobserver and interobserver reproducibility for atrial strain and SR parameters, and fair to good for LA booster strain. These results are in line with a previous research by Dick et al.^15^ Reproducibility for RA strain was poorer than LA strain, likely because of RA measurements in one view only (4-chamber) compared with LA measurements, which were done in 3 different views (3-chamber, 2-chamber, and 4-chamber).

A major limitation of this research is the relatively small number of patients and the retrospective selection of the patients’ cohort. However, we enrolled exclusively TS patients with the apical type. The promising results of our study prompt further prospective trials including larger cohorts to confirm our findings. Moreover, the predictive value of atrial strain for adverse cardiovascular events has not been assessed in our study at the follow-up. Finally, the impairment in atrial strain in patients with TS would have been probably different if CMR was performed within a shorter period of time, ideally the same day of hospital admission.

In this study, patients with TS showed significantly lower LA reservoir and conduit functions compared with the AM group. Our study findings suggest that LA impairments can be an additional quantitative parameter to discriminate TS and AM, helping clinicians in the challenging differential diagnosis of these 2 entities.

## References

[1] Ghadri J-R Wittstein IS Prasad A . International Expert Consensus Document on Takotsubo Syndrome (Part I): clinical characteristics, diagnostic criteria, and pathophysiology. Eur Heart J. 2018;39:2032–2046. 29850871 10.1093/eurheartj/ehy076 PMC5991216

[2] Ono R Falcão LM . Takotsubo cardiomyopathy systematic review: pathophysiologic process, clinical presentation and diagnostic approach to Takotsubo cardiomyopathy. Int J Cardiol. 2016;209:196–205. 26896623 10.1016/j.ijcard.2016.02.012

[3] Stiermaier T Graf T Möller C . Transient left atrial dysfunction is a feature of Takotsubo syndrome. J Cardiovasc Magn Reson. 2017;19:15. 28162089 10.1186/s12968-017-0328-8 PMC5292816

[4] Akashi YJ Nef HM Lyon AR . Epidemiology and pathophysiology of Takotsubo syndrome. Nat Rev Cardiol. 2015;12:387–397. 25855605 10.1038/nrcardio.2015.39

[5] Cau R Bassareo P Cherchi V . Early diagnosis of chemotherapy-induced cardiotoxicity by cardiac MRI. Eur J Radiol. 2020;130:109158. 32652404 10.1016/j.ejrad.2020.109158

[6] Bossone E Lyon A Citro R . Takotsubo cardiomyopathy: an integrated multi-imaging approach. Eur Hear J Cardiovasc Imaging. 2014;15:366–377. 10.1093/ehjci/jet167 24128655

[7] Lyon AR Bossone E Schneider B . Current state of knowledge on Takotsubo syndrome: a position statement from the Taskforce on Takotsubo Syndrome of the Heart Failure Association of the European Society of Cardiology. Eur J Heart Fail. 2016;18:8–27. 10.1002/ejhf.424 26548803

[8] Madhavan M Prasad A . Proposed Mayo Clinic criteria for the diagnosis of Tako-Tsubo cardiomyopathy and long-term prognosis. Herz. 2010;35:240–244. 20582391 10.1007/s00059-010-3339-x

[9] Scatteia A Baritussio A Bucciarelli-Ducci C . Strain imaging using cardiac magnetic resonance. Heart Fail Rev. 2017;22:465–476. 28620745 10.1007/s10741-017-9621-8 PMC5487809

[10] Williams LK Forero JF Popovic ZB . Patterns of CMR measured longitudinal strain and its association with late gadolinium enhancement in patients with cardiac amyloidosis and its mimics. J Cardiovasc Magn Reson. 2017;19:61. 28784140 10.1186/s12968-017-0376-0 PMC5545847

[11] Cau R Bassareo P Deidda M . Could CMR tissue-tracking and parametric mapping distinguish between Takotsubo syndrome and acute myocarditis? A pilot study. Acad Radiol. 2021. In press. 10.1016/j.acra.2021.01.009 33487539

[12] Truong VT Palmer C Young M . Right atrial deformation using cardiovascular magnetic resonance myocardial feature tracking compared with two-dimensional speckle tracking echocardiography in healthy volunteers. Sci Rep. 2020;10:1–7. 32251322 10.1038/s41598-020-62105-9 PMC7089993

[13] Truong VT Palmer C Wolking S . Normal left atrial strain and strain rate using cardiac magnetic resonance feature tracking in healthy volunteers. Eur Heart J Cardiovasc Imaging. 2020;21:446–453. 31504357 10.1093/ehjci/jez157

[14] Hinojar R Zamorano JL Fernández-Méndez MA . Prognostic value of left atrial function by cardiovascular magnetic resonance feature tracking in hypertrophic cardiomyopathy. Int J Cardiovasc Imaging. 2019;35:1055–1065. 30706353 10.1007/s10554-019-01534-8

[15] Dick A Schmidt B Michels G . Left and right atrial feature tracking in acute myocarditis: a feasibility study. Eur J Radiol. 2017;89:72–80. 28267553 10.1016/j.ejrad.2017.01.028

[16] Doerner J Bunck AC Michels G . Incremental value of cardiovascular magnetic resonance feature tracking derived atrial and ventricular strain parameters in a comprehensive approach for the diagnosis of acute myocarditis. Eur J Radiol. 2018;104:120–128. 29857857 10.1016/j.ejrad.2018.05.012

[17] Caforio ALP Pankuweit S Arbustini E . Current state of knowledge on aetiology, diagnosis, management, and therapy of myocarditis: a position statement of the European Society of Cardiology Working Group on Myocardial and Pericardial Diseases. Eur Heart J. 2013;34:2636–2648; 2648a–2648d. 23824828 10.1093/eurheartj/eht210

[18] Cau R Bassareo P Caredda G . Atrial strain by feature-tracking cardiac magnetic resonance imaging in Takotsubo cardiomyopathy. features, feasibility, and reproducibility. Can Assoc Radiol J. 2021. [Epub ahead of print]. 10.1177/08465371211042497 34615401

[19] Eitel I Lücke C Grothoff M . Inflammation in takotsubo cardiomyopathy: insights from cardiovascular magnetic resonance imaging. Eur Radiol. 2010;20:422–431. 19705125 10.1007/s00330-009-1549-5

[20] Stiermaier T Lange T Chiribiri A . Right ventricular strain assessment by cardiovascular magnetic resonance myocardial feature tracking allows optimized risk stratification in Takotsubo syndrome. PLoS One. 2018;13:e0202146. 30157266 10.1371/journal.pone.0202146 PMC6114723

[21] Stiermaier T Lange T Chiribiri A . Left ventricular myocardial deformation in Takotsubo syndrome: a cardiovascular magnetic resonance myocardial feature tracking study. Eur Radiol. 2018;28:5160–5170. 29882071 10.1007/s00330-018-5475-2

[22] Cau R Bassareo PP Mannelli L . Imaging in COVID-19-related myocardial injury. Int J Cardiovasc Imaging. 2021;37:1349–1360. 33211242 10.1007/s10554-020-02089-9 PMC7676417

[23] Ghadri J-R Wittstein IS Prasad A . International Expert Consensus Document on Takotsubo Syndrome (Part II): diagnostic workup, outcome, and management. Eur Heart J. 2018;39:2047–2062. 29850820 10.1093/eurheartj/ehy077 PMC5991205

[24] Abhayaratna WP Seward JB Appleton CP . Left atrial size: physiologic determinants and clinical applications. J Am Coll Cardiol. 2006;47:2357–2363. 16781359 10.1016/j.jacc.2006.02.048

[25] Schuster A Backhaus SJ Stiermaier T . Impact of right atrial physiology on heart failure and adverse events after myocardial infarction. J Clin Med. 2020;9:210. 31940959 10.3390/jcm9010210 PMC7019524

[26] Backhaus SJ Stiermaier T Lange T . Atrial mechanics and their prognostic impact in Takotsubo syndrome: a cardiovascular magnetic resonance imaging study. Eur Hear J Cardiovasc Imaging. 2019;20:1059–1069. 10.1093/ehjci/jey219 30649241

